# The Danish Multiple Sclerosis Registry

**DOI:** 10.1002/brb3.1921

**Published:** 2020-10-30

**Authors:** Melinda Magyari, Hanna Joensen, Bjarne Laursen, Nils Koch‐Henriksen

**Affiliations:** ^1^ Department of Neurology The Danish Multiple Sclerosis Registry Rigshospitalet Glostrup Denmark; ^2^ Danish Multiple Sclerosis Center Department of Neurology Rigshospitalet Glostrup Denmark; ^3^ National Institute of Public Health University of Southern Denmark Copenhagen Denmark; ^4^ Department of Clinical Epidemiology Aarhus University Hospital Aarhus Denmark

**Keywords:** data source, epidemiology, multiple sclerosis, observational studies, real world data, registry

## Abstract

**Objectives:**

The Danish Multiple Sclerosis Registry is the oldest operative and nationwide MS registry. We present The Danish Multiple Sclerosis Registry with its history, data collection, scientific contribution, and national and international research collaboration.

**Materials and Methods:**

Detailed description of data collection, completeness, quality optimizing procedures, funding, and legal, ethical and data protection issues are provided.

**Results:**

The total number of registered cases with clinical isolated syndrome and multiple sclerosis since 1956 was by start of May 2020 30,023 of whom 16,515 cases were alive and residing in Denmark, giving a prevalence rate of about 284 per 100,000 population. The mean annual number of new cases receiving an MS diagnosis was 649 per year in the period 2010 to 2019. In total, 7,945 patients (48.1%) are receiving disease modifying therapy at the start of May 2020.

**Conclusions:**

Multiple Sclerosis registers are becoming increasingly important, not only for epidemiological research but also by quantifying the burden of the disease for the patients and society and helping health care providers and regulators in their decisions. The Danish Multiple Sclerosis Registry has served as data source for a number of scientific publications including epidemiological studies on changes in incidence and mortality, cohort studies investigating risk factors for developing MS, comorbidities and socioeconomic outcomes in the MS population, and observational studies on effectiveness of disease modifying treatments outside the narrow realms of randomized clinical trials.

## Graphical abstract

We present The Danish Multiple Sclerosis Registry with its history, data collection, scientific contribution, and national and international research collaboration. The Danish Multiple Sclerosis Registry has served as data source for a number of scientific publications, epidemiological studies on changes in incidence and mortality, cohort studies investigating risk factors for developing MS, comorbidities and socioeconomic outcomes in the MS population and observational studies on effectiveness of disease modifying treatments outside the narrow realms of randomised clinical trials.

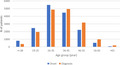

## 1 INTRODUCTION

Registries are powerful data sources. Observational studies of multiple sclerosis (MS) derived from registries are increasingly important contributors to epidemiology, to natural history of MS including survival, and to “real‐world” evidence of effectiveness and safety of disease modifying drugs (DMD). A large number of MS registers have been established within recent decades. A search in PubMed for articles with the terms “multiple sclerosis” and “register” or “registry” in singular and plural forms in the title revealed only 9 articles before 2000, 31 articles in the 2000s and 166 articles from 2010 to date. The nationwide Danish Multiple Sclerosis Registry is among the oldest and largest registers of MS under continuous development and update. With this article, we aim at describing its history, contents, and methods, as it is the background for much of the Danish MS research.

## 2 MATERIALS AND METHODS

### 2.1 History

The Danish Multiple Sclerosis Registry (DMSR) (Koch‐Henriksen et al., 2001) was formally established in 1956, but prospective data collection started already in 1948. It commenced as a nationwide population‐based MS prevalence survey (Hyllested, 1956) and has since continued to register new cases of MS. This register was set up and suited for descriptive and analytical MS epidemiology and survival analyses.

The target population of the DMSR is all Danish citizens, who have received a diagnosis of MS by a neurologist or a Department of Neurology (including the two Danish MS Rehabilitation Hospitals). Discharge letters or copies of the clinical records were sent to the DMSR until 2015, replaced by an online data collection system.

Disease modifying therapy (DMT) for patients with relapsing remitting multiple sclerosis (RRMS) became available in Denmark in 1996. It was from start mandatory to follow all treated patients at least once or twice a year and report data on side effects and current disease activity to a central register, the newly established Danish Multiple Sclerosis Treatment Register (DMSTR), (Koch‐Henriksen & Sorensen, 2000; Magyari et al., 2016) which is associated with the DMSR.

From 2007, the DMSTR was by the health authorities classified as a national clinical quality database with the aim of monitoring and improving the quality and safety of DMT of patients with MS. DMSTR is suited for clinical follow‐up studies, but it only included patients receiving DMT.

In 2015, the DMSR established a new data collection platform, COMPOS® (Hillert & Stawiarz, 2015) which is now the common online data collection software for both the DMSR and for the clinical quality register DMSTR. The DMSR serves scientific purposes as it comprises all collected information including treatment with DMT on the entire Danish MS population, while the DMSTR itself remained a clinical quality database, ensuring a high quality of MS care in the clinics. A yearly report is published in collaboration with The Danish Clinical Quality Program—National Clinical Registries (https://www.rkkp.dk). Treating neurologists from all neurological departments are joined in a network, the Danish Multiple Sclerosis Group (DMSG), which meets once or twice a year to discuss and establish uniform guidelines. From this follows a virtually complete and regular registration of clinical disease activity. The clinicians in the DMSG meet regularly and determine indicators and standards for good clinical quality, and recommendations are reported back to clinical personnel in writing for quality improvement and released in public for transparency and accountability.

### 2.2 Data collection

The DMSR collects data on from the whole country on patients with the demyelinating conditions: MS, radiological isolated syndrome (RIS), and clinical isolated syndrome (CIS). We have extended the target of the DMSR to include the other demyelinating disease, neuromyelitis optica spectrum disorder (NMOSD). Data on the population with NMOSD were added retrospectively after a recent nationwide incidence and prevalence survey going back to 2007 (Papp et al., 2018).

In Denmark, far most of the neurological services are performed by the public hospitals. Diagnostic and clinical management of patients with MS and related disorders are carried out by 13 Departments of Neurology/MS clinics in public hospitals, and these are the only units that are authorized to prescribe and dispense disease modifying drugs.

All treating clinics record data online into COMPOS® on all patients with any of the above‐mentioned demyelinating diseases. Notification starts when the disease is diagnosed; however, much attention is paid to anamnestic clarification of the time of clinical disease onset. During treatment, patients are monitored in connection with scheduled clinical visits at regular intervals with recording of demographic, clinical and paraclinical data, as well as Kurtzkes Expanded Disability Status Score (EDSS) and functional systems (FS) (Kurtzke, 1983), and side effects. Relapses are recorded with their dates and whether or not treatment with corticosteroid was applied. Notification of the DMSR is mandatory for all patients receiving DMT, and data collection is a part of the routine medical follow‐up of patients. Failure of reporting mandatory data to the DMSR for a department will be observed by the health authorities who are the owners of the Danish public hospitals.

Prospective active recording of adverse drug reactions or adverse events (AE), as part of the minimum dataset, became possible upon expansion of the online data collection platform in May 2018. An active assessment of AEs takes place at each clinical visit and if a patient reports a new AE since the last visit, a detailed adverse event module must be filled in. All recorded AEs since May 2018 are converted into ICD‐10 codes.

The online data collection platform facilitates the implementation of additional data modules required by specific studies, for example, a rapid implementation of data collection on Covid‐19 cases in the spring of 2020.

Because notification on all patients with MS treated with DMD is mandatory, data on patients receiving DMD are virtually complete.

### 2.3 Legal context, ethics, and data protection

All patients are informed about their data being recorded. According to the Danish implementation of the General Data Protection Regulation (GDPR), there is no requirement for informed consent from the patient for inclusion in the DMSR, on the explicit condition that data can never be used for any other purpose than pure science and statistics. The hospital owners and relevant councils are provided with summary counts of patients to estimate future needs for DMDs and medical care.

The DMSR is physically adjoined to the Department of Neurology, University Hospital Copenhagen, Rigshospitalet.

### 2.4 Data usage rights, data‐sharing, data protection and data privacy

The DMSR is a scientific research register and data cannot be accessed for other purposes. The Scientific Steering Committee of the DMSR reviews and assesses the research proposals according to scientific quality, feasibility, value of the project, and alignment with priority areas of the scientific activities of the DMSR.

After evaluation of the application by the Scientific Committee, a contract will be drawn up for each project, setting out the rules of data property and budget aspects. Access to data will then be provided. Data are only available to academics after specific permission and limited to what is necessary for the purpose of then defined scientific project. Data analysis is also carried out by DMSR or an independent third party according to GDPR regulations.

## 3 RESULTS

### 3.1 Number of cases registered

The total number of registered with CIS and MS was by start of May 2020 30,023 of whom 16,515 cases were alive and residing in Denmark, giving a prevalence rate of about 284 per 100,000 population. Details of the register content of these patients are shown in Table 1, and the variables are listed in Table 2. The mean annual number of new cases receiving an MS diagnosis was 649 per year in the period 2010 to 2019 corresponding to an incidence rate of 11.46 per 100,000 person years (95% CI 10.57–12.35). In total, 7,945 patients (48.1%) are receiving DMT at the start of May 2020. An annual report in Danish is drafted of all patients included in the DMSR to provide regular descriptions of the Danish MS population.

**Table 1:** Number of cases first diagnosed since January 1, 1996, with multiple sclerosis and clinical isolated syndrome. Snapshot of data as of May 2020

Diagnosis^a^	Prevalent cases^b^	Cases with at least one EDSS report	Patients with at least one relapse report	Total number of EDSS reports	Total number of relapse reports	Total number of MRI reports	Total number of DMT starts	Total number of DMT discontinuations	Total number of ongoing DMT (patients)	Mean follow up time (years) ^c^
CIS	266	223	73	1,044	75	604	205	102	103	6
MS (without specified disease course)	1,089	842	191	2,005	311	1,349	463	143	321	7
RRMS	8,972	8,898	6,134	109,819	20,129	39,131	20,229	13,326	6,810	10
PPMS	534	452	44	1,486	76	851	92	31	60	12
SPMS	1,425	1,383	809	19,602	2,987	3,871	2,493	2,026	470	15

^a^ Latest diagnosis. A patient may have more than one MS‐related diagnosis over time.

^b^ Cases receiving first MS‐related diagnosis from January 1996 to May 2020.

^c^ From diagnosis to death, emigration, termination or day of data extraction.

**Table 2:** Main variables of the DMSR

Patient	MS history	Laboratory tests	Relapses	MRI	Disability	Disease modifying therapy	Adverse events
Demographic characteristics	From onset to diagnosis and disease course	Tests upon diagnosis and onwards	Relapses during DMT	MRI of brain and/or spinal cord from diagnosis onwards	EDSS assessed at regular clinical visits	Treatment sequences	Adverse Events (AE) suspected related to DMT
Date of birth	Date of onset	Date of test	Date of relapse	Date of MRI	Date of test	Treatment start date	Date of AE
Gender	Onset symptoms	IgG index	Steroid treatment	*N* of T2 lesions	Functional System Score (each function)	Treatment end date	ICD 10 code
	Relapses 24 months prior to diagnosis	Oligoclonal bands		*N* of new T2 lesions	EDSS score	Treatment drug	Seriousness
	Date of diagnosis	Antibodies (NAbs, Anti Tysabri)		*N* of Gadolinium enhancing lesions		Reasons for discontinuation	Outcome of AE
	Disease course/ conversion	JCV					Consequences of AE on DMT continuation

Note

Abbreviations: DMT, Disease Modifying Therapy; EDSS, Expanded Disability Status Scale; JCV, John Cunningham Virus; NAbs, neutralizing antibodies against interferon‐β.

**FIGURE 1:**
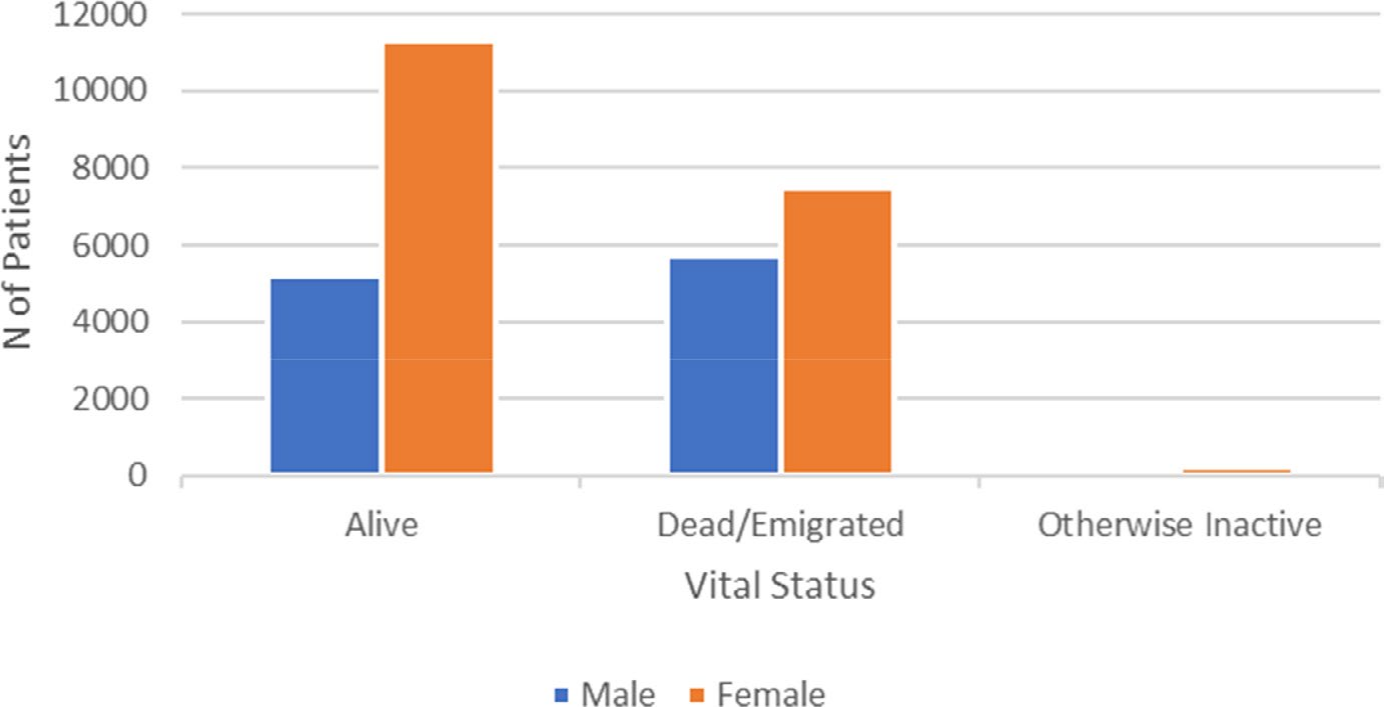
Total population of patients with MS and CIS. Distribution by vital status and sex

**FIGURE 2:**
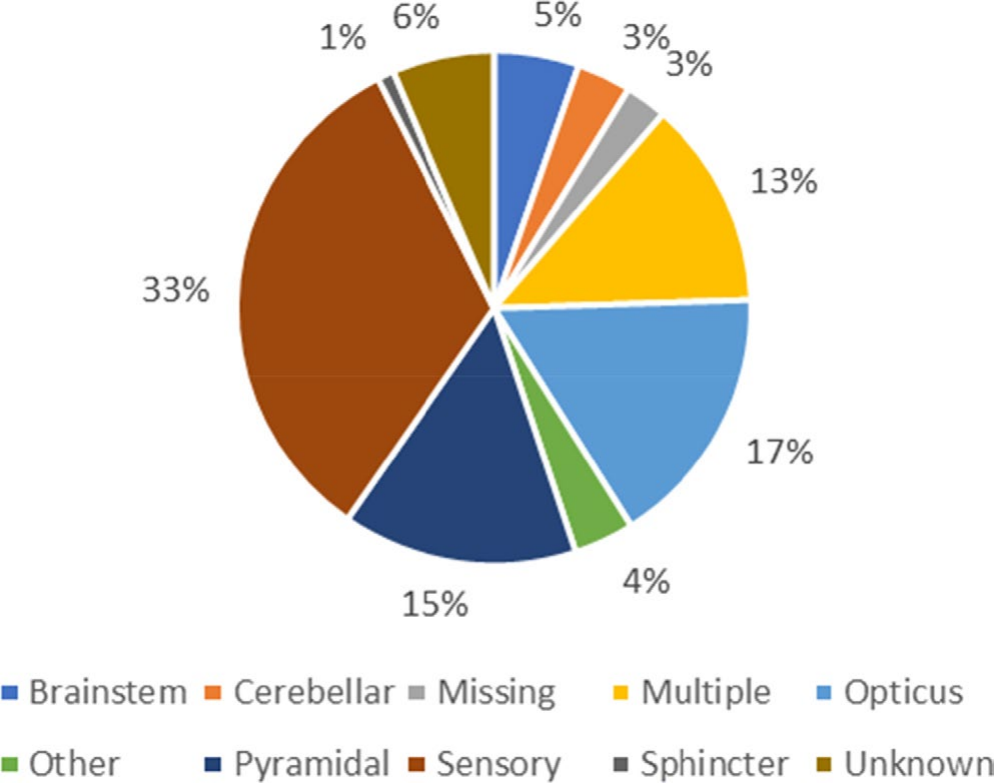
Onset symptom for the prevalent population with MS and CIS

**FIGURE 3:**
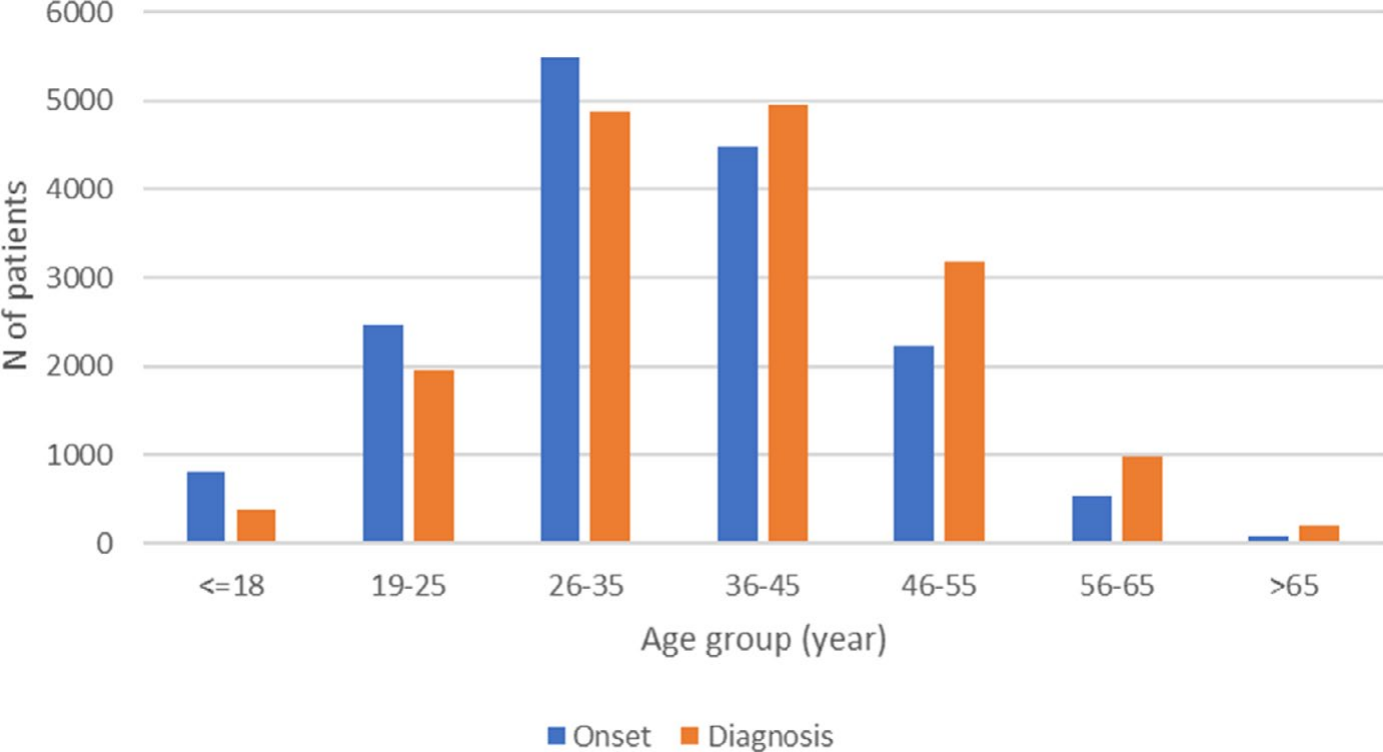
Age group at onset and diagnosis for the prevalent MS and CIS population

**FIGURE 4:**
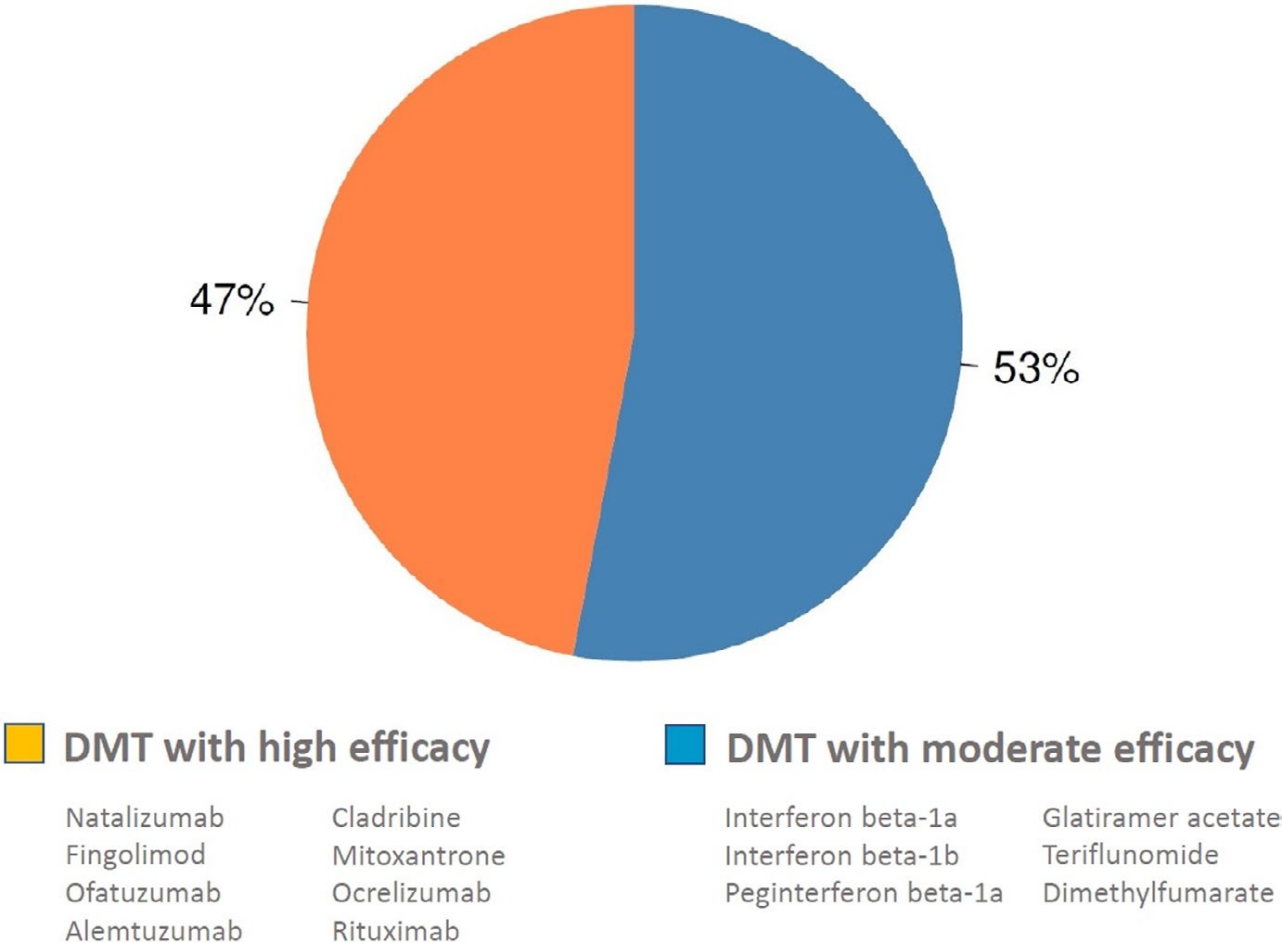
Distribution of ongoing disease modifying therapy by May 2020

The treatment history for patients has been registered since 1996, and side effect from treatment has been reported from the beginning, though more detailed since 2018. Relapses, EDSS, and MRI have been regularly reported after treatments became available and more detailed in recent years. Figure 1 shows the distribution of the patients as to vital status and sex; Figure 2 shows the distribution of onset symptom; Figure 3 shows age at onset and age at diagnosis. Current treatment by type of preparation DMT is presented in Figure 4.

### 3.2 Optimizing quality of the DMSR

#### 3.2.1 Validity

In the old part of the DMSR, which was based on submitted clinical records or discharge letters, the neurological staff of the register evaluated all clinical data as to compliance with the current diagnostic criteria (until 1994 those of (Allison & Millar, 1954) from 1994 the Poser criteria (Poser et al., 1983); and from 2005 the McDonald criteria (Polman et al., 2005)). Only three neurologists (one of them NKH) have since 1948 been involved in this task.

**FIGURE 5:**
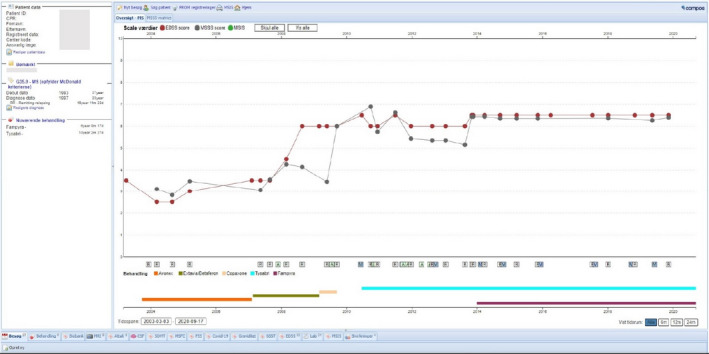
The COMPOS platform for data entry, helping the doctor with an overview of prior relapses, treatment, and disability

After 2015, the reporting neurologists from the departments of neurology have been responsible for application of the current diagnostic criteria. Each department has online access to an overview of the patients they have reported, supporting their daily clinical work. This facilitates the neurologists’ interest in recording high‐quality homogeneous data in the online system and ensures the scientific validity of the registered data. Additional quality control is performed ensuring correct format and structure of data, the plausibility and consistency of data within a data set and within longitudinal data of one patient (Figure 5). The data collection software has an integrated data verification tool to identify missing or incoherent data. The assessment of EDSS and FS is performed in the departments by the neurologists for whom the definitions of FS and EDSS were always at hand: either on the rear side of the score sheet or later as a drop‐down menu. Data are normally entered directly into the COMPOS system by the doctor and, if not, by the MS nurse, based on the doctors’ record. The DMSTR provides monthly feedback to the reporting clinics on the selected quality indicators which further enhances the quality of reporting to the benefit of DMSR.

#### 3.2.2 Completeness

The DMSR is regularly linked with other registers: As a source of control of the completeness, we use the Danish National Patient Registry (NPR) (Schmidt et al., 2015). This registry is automatically notified by all Danish hospitals of all in‐ and outpatient contacts by person identification, dates of contact, and ICD‐10 (former ICD‐8) diagnoses. The completeness and validity of the NPR with reference to the DMSR proved to be 86.9% and 96.3%, respectively (Mason et al., 2012).

The Danish Civil Registration System (Pedersen, 2011) provides all Danish citizens who were alive in 1968 and thereafter with a unique 10‐digit person ID (CPR), the first six digits being the date of birth, the last digit indicating the gender. This enables linkage with other registers and provides the DMSR with complete vital status and dates of death or emigration, and it prevents double registration because the CPR is used as the primary ID of the patient in COMPOS®.

To further ensure completeness of the DMSR, the data coordinator of the DMSR receives monthly lists of all patients assigned to any of the MS clinics, independent of the notifications by COMPOS, and control of correct case‐definition has a high priority for the DMSR. The advantage for the doctors is that the COMPOS platform for the single patients provides an instantaneous overview of the course of relapses, EDSS, MSSS, treatments, and side effects (Figure 5). This contributes to a high completeness of registration.

### 3.3 Scientific contribution

The DMSR has alone or in collaboration with other scientists published more than 130 scientific papers in the fields of (a) descriptive epidemiology; (b) historical prospective studies in which sub‐populations subjected to different potential putative risk factors have been compared with the background population as to later emergence of MS; (c) MS cohort observational studies of clinical endpoints or mortality and the influence of demographic or clinical factors and different exposures; (d) case–control studies comparing different early‐life exposures in MS cases and background population controls; (e) head‐to‐head treatment comparison studies.

We have selected most important studies from the DMSR published within the last 10 years.

#### 3.3.1 Descriptive epidemiology

- Incidence of MS has increased markedly, particularly in elderly and in women (Koch‐Henriksen et al., 2018).
- Incidence of NMOSD in Denmark was estimated at 0.029 per 100,000 person years (Papp et al., 2018).
- The incidence rates of pediatric MS and other ADS in Denmark were higher than those reported for some other European countries (Boesen et al., 2018).
- Spatial analyses revealed that even within the small geographical area of Denmark, there were significant variations in MS incidence (Bihrmann et al., 2018).

#### 3.3.2 Historical prospective population cohort studies

- Low concentrations of neonatal 25(OD) vitamin D increase the risk of MS later in life (Nielsen et al., 2017).
- Shift work at age 15–19 increases the risk of MS in adulthood (Gustavsen et al., 2016).
- MS seems to considerably impact reproductive choices (Moberg et al., 2019).
- Immigrants from low‐MS‐risk countries only partially adapted to the risk in Denmark, most pronounced with immigration before age 15 (Nielsen et al., 2019).
- Early age at menarche appears to be associated with an increased risk of MS (Nielsen et al., 2017).
- Socioeconomic status in childhood seems of no major importance for the subsequent risk of MS. (Nielsen et al., 2013)
- People born by Cesarean section were at no increased risk of MS later in life (Nielsen et al., 2013).
- Use of antibiotics increases the risk of MS suggesting that the underlying infections may be causally associated with MS (Norgaard et al., 2011).
- High body mass index in childhood is a risk factor for MS (Munger et al., 2013).
- Male infertility increases the risk of MS (Glazer et al., 2017).
- Parental MS affects educational achievements, employment, disability pension, and income of children (Moberg et al., 2016).

#### 3.3.3 MS cohort observational studies

- Relapses worsen disability significantly beyond the recovery phase (Koch‐Henriksen et al., 2019).
- Surviving times in patients with MS have improved steadily over several decades (Koch‐Henriksen et al., 2017).
- The efficacy of DMT in pediatric onset MS patients is comparable to that seen in adult onset MS patients. (Kopp et al., 2019)
- Earlier treatment start is associated with a beneficial prognosis in the pediatric cohort (Kopp et al., 2019).
- Patients who started treatment with DMT later reached an EDSS score of 6 more quickly compared with patients who started early, and the delay showed a tendency to shorten time to death (Chalmer et al., 2018).
- Multiple sclerosis patients do not have increased cancer incidence or increased cancer‐specific mortality compared with the background population (Norgaard et al., 2018).
- Compared with the background population MS seriously affects the economic life of the patients, even within a few years of onset (Pfleger et al., 2010), and it often leads to broken relationships (Pfleger et al., 2010).
- Treatment effectiveness of Interferon‐β is not different in women and men (Magyari et al., 2014).
- Clinically stable disease is associated with a lower risk of both income loss and disability pension for patients with multiple sclerosis (Chalmer et al., 2020).
- Treatment escalation leads to fewer relapses compared with lateral switching to another moderately effective therapy (Chalmer et al., 2019).
- The occurrence of vascular comorbidities is higher in the population with MS (Thormann et al., 2016).
- Comorbidities in multiple sclerosis have socioeconomic consequences and are associated with diagnostic delays and increased mortality (Magyari et al., 2014).

#### 3.3.4 Case‐control studies

- Female MS patients have fewer childbirths than women from the matched background population in the last five years up to clinical onset (Magyari et al., 2013).
- Social and physical environmental factors available from Danish registries cannot explain the increasing incidence of women in Denmark (Magyari et al., 2014).
- Some autoimmune comorbidities are more frequent in persons with MS (Magyari et al., 2014).

#### 3.3.5 Head‐to‐head comparative treatment studies

- Reduced disease activity in MS patients treated with dimethyl fumarate compared with patients treated with teriflunomide (Buron et al., 2019).
- Natalizumab and fingolimod had equal clinical effect in RRMS (Koch‐Henriksen et al., 2017).

## 4 DISCUSSION

The DMSR has proved valuable as a scientific registry and has gained increasing attention through the last decades. The advantages of the DMSR are that it is close to being purely population based, and that its size in many instances secures a substantial statistical power. The scientific questions typically address range from purely descriptive questions aimed at understanding the characteristics of people who develop the disease and how the disease generally progresses, to highly focused questions intended to support decision‐making on disease modifying therapy. The most important questions about registers are their completeness and validity, which can have different weights depending on the type of a study. We have aimed to achieve optimal completeness by the independent case ascertainment method using multiple sources of notification, and at our best ensured validity by the diagnostic criteria method (Goldberg et al., 1980).

Several studies have been published by linking the DMSR to numerous other Danish population‐based registers, carrying person‐specific information on vast variety of clinical, social, health, financial, educational, and occupational data.

The DMSR is involved in international collaborations and is a part of the BIGMS Network which is a network of five MS patient registries, with the joint aim to analyze large‐scale information on MS. Several past and ongoing scientific projects are conducted in collaboration with national or international research groups using the DMSR as data source.

The DMSR is also a resource for post‐marketing authorization safety and effectiveness studies (i.e., phase IV), which can be conducted by utilizing its prospectively collecting safety data and linkage to independently collected secondary data information from several population‐based registries, such as The National Patient Registry (Schmidt et al., 2015), The National Prescription Database (Johannesdottir et al., 2012), The Registry of causes of death (Helweg‐Larsen, 2011), and The Cancer Registry (Gjerstorff, 2011). Five phase IV studies are in progress regarding post‐marketing safety of teriflunomide, alemtuzumab, cladribine, natalizumab, and ocrelizumab. Furthermore, by linkage of the DMSR to nationwide population based reproductive registries we are able to evaluate the association between exposure to a specific drug and different pregnancy and perinatal outcomes. Currently, the DMSR is involved in several multi‐country cohort post‐authorization pregnancy safety studies. Such studies are also conducted at national level if the sample size allows a sufficient statistical power.

### 4.1 Ongoing efforts

In 2020, the data collection platform will be expanded with a new module to enable self‐reported information from people with MS, known as patient‐reported outcomes (PRO). An ongoing project in collaboration with the Danish MS Society aims to ensure patient participation in the selection of specific scales, surveys and outcomes, which will be a part of the online PRO module.

Specific modules can be implemented in order to expand the collected data or if the need to collect specific information arises. For example, a module to collect data on Covid‐19 infection has been implemented very fast during April 2020. The core COVID‐19 dataset will contribute to a global data sharing initiative and can serve to answer questions on the impact of the Covid‐19 pandemic on different aspects of MS.

The possibility to adjust the data collection platform according to emerging research questions is of increasing importance for patients, researchers as well as for health authorities and industrials and is a step forward toward personalized patient management.

## 5 CONCLUSION

The DMSR has served as data source for a number of scientific publications, epidemiological studies on changes in incidence and mortality, cohort studies investigating risk factors for developing MS, comorbidities and socioeconomic outcomes in the MS population and observational studies on effectiveness of disease modifying treatments outside the narrow realms of randomized clinical trials.

## CONFLICT OF INTERESTS

The authors received no financial support for the authorship, and publication of this article. Melinda Magyari has served on scientific advisory board for Biogen, Sanofi, Roche, Novartis, Merck, Abbvie, has received honoraria for lecturing from Biogen, Merck, Novartis, Sanofi, Genzyme, has received research support and support for congress participation from Biogen, Genzyme, Roche, Merck, Novartis. Hanna Joensen received honoraria for participating in advisory board from Biogen. Bjarne Laursen has nothing to disclose. Nils Koch‐Henriksen has received support for participation in congresses and symposia by Biogen, Merck, Novartis, Sanofi Genzyme, and Teva, and has received fee for lecturing by Novartis.

## AUTHOR CONTRIBUTIONS

Melinda Magyari conceptualized the article, has been involved in drafting and revising the manuscript. Hanna Joensen performed data acquisition and analyses and revised the manuscript. Bjarne Laursen assisted in data acquisition and revising the manuscript. Nils Koch‐Henriksen conceptualized the article, has been involved in drafting and revising the manuscript.

### Peer Review

The peer review history for this article is available at https://publons.com/publon/10.1002/brb3.1921.

## Data Availability

Data will be shared upon request by any qualified investigator under approval from the Danish Data Protection Agency.
